# Readability assessment of patient educational materials for pediatric spinal deformity from top academic orthopedic institutions

**DOI:** 10.1007/s43390-022-00545-1

**Published:** 2022-07-11

**Authors:** Christopher Michel, Christopher Dijanic, George Abdelmalek, Suleiman Sudah, Daniel Kerrigan, George Gorgy, Praveen Yalamanchili

**Affiliations:** 1 Department of Orthopedic Surgery, Monmouth Medical Center-RWJBarnabas Health, Long Branch, NJ 07740 USA; 2 Rutgers New Jersey Medical School, Newark, NJ 07103 USA

**Keywords:** Pediatric, Deformity, Patient-education, Reading

## Abstract

**Study design:**

Cross-sectional analysis of patient educational materials from top pediatric orthopedic hospital websites.

**Objective:**

To assess the readability of online educational materials of top pediatric orthopedic hospital websites for pediatric spinal deformity.

**Summary of background data:**

The internet has become an increasingly popular source of health information for patients and their families. Healthcare experts recommend that the readability of online education materials be at or below a 6^th^-grade reading level. However, previous studies have demonstrated that the readability of online education materials on various orthopedic topics is too advanced for the average patient. To date, the readability of online education materials for pediatric spinal deformity has not been analyzed.

**Methods:**

Online patient education materials from the top 25 pediatric orthopedic institutions, as ranked by the U.S. News and World Report hospitals for pediatric orthopedics, were accessed utilizing the following readability assessments: Flesch–Kincaid (FK), Flesch Reading Ease, Gunning Fog Index, Coleman–Liau Index, Simple Measure of the Gobbledygook Index (SMOG), Automated Readability Index, FORCAST, and the New Dale and Chall Readability. Correlations between academic institutional ranking, geographic location, and the use of concomitant multi-media modalities with FK scores were evaluated using a Spearman regression.

**Results:**

Only 48% (12 of 25) of top pediatric orthopedic hospitals provided online information regarding pediatric spinal deformity at or below a 6^th^-grade reading level. The mean FK score was 9.0 ± 2.7, Flesch Reading Ease 50.8 ± 15.6, Gunning Fog Score 10.6 ± 3.1, Coleman–Liau Index 11.6 ± 2.6, SMOG index 11.7 ± 2.0, Automated Readability Index 8.6 ± 2.8, and Dale–Chall Readability Score 6.4 ± 1.4. There was no significant correlation between institutional ranking, geographic location, or use of multimedia with FK scores.

**Conclusion:**

Online educational material for pediatric spinal deformity from top pediatric orthopedic institutional websites are associated with poor readability.

## Introduction

The internet has become a widely used resource for patients seeking orthopedic information. Over 8 million Americans use the internet each day to learn about their health conditions and treatment options [1–6]. Parents, in particular, rate online health information as one of their most valuable tools, with an estimated 98% having used the internet to search for information regarding the health of their child [7]. However, the use of medical terminology and complex writing styles may limit comprehension of online educational materials amongst patients and parents of different educational backgrounds. Recent recommendations from the National Institutes of Health and the National Academy of Medicine recommend that patient educational materials should be written at the sixth-grade reading level or below [1, 2, 8–12].

Readability is a numerical value that is determined by systematic formulas, reflecting the grade level of reading necessary to understand the information [13, 14]. The Flesch-Kincaid reading level (FK) is a popular readability score that was created by the U.S. military and has been validated in previous studies [15, 16]. Other reading scores that emphasize different metrics compared to FK include the Coleman–Liau Index, New Dale–Chall Readability Formula, FORCAST Readability Formula, Gunning Fog Index, Simple Measure of Gobbledygook (SMOG), and Automated Readability Index. The formulas for these scores are found in Table 1. Investigators have studied the readability of patient education materials across various orthopedic subspecialties including adult reconstruction [13], foot and ankle [14], shoulder and elbow [11, 12], spine [3], hand [4], arthroscopy [17], sports medicine [10, 18], pediatric orthopedics, and the American Academy of Orthopedics website itself [1]. However, to our knowledge, no study has evaluated the readability of online patient education materials regarding pediatric spinal deformity.

**Table 1:** Formulas for readability metrics

Readability assessment	Formula
Flesch–Kincaid	(0.39 × mean # of syllables per word)) + (11.8 × mean # of words per sentence)
Flesch Reading Ease	206.835 − (1.015 × mean # of words per sentence) − (84.6 × mean # of syllables per word)
Gunning Fog Index	$0.{4} \times \left( {\frac{{{\text{mean}}\;\# \;{\text{of}}\;{\text{words}}}}{{{\text{mean}}\;\# \;{\text{of}}\;{\text{sentences}}}} + 100 \times \left( {\frac{{{\text{mean}}\;\# \;{\text{of}}\;{\text{words}}\;{\text{with}}\; \ge 3\;{\text{syllables}}}}{{{\text{mean}}\;\# \;{\text{of}}\;{\text{words}}}}} \right)} \right)$
Coleman–Liau Index	$\left( {0.0588 \times \frac{{{\text{mean}}\;\# \;{\text{of}}\;{\text{letters}}}}{{{\text{word}}}} - \left( {0.296 \times \frac{{{\text{mean}}\;\# \;{\text{of}}\;{\text{sentences}}}}{{100\;{\text{words}}}}} \right)} \right)$
Simple measure of the Gobbledygook (SMOG) Index	$1.043 \times \sqrt {(\# \;{\text{of}}\;{\text{words}}\;{\text{with}}\; \ge 3\;{\text{syllables}}) \times \left( {\frac{30}{{\# \;{\text{of}}\;{\text{sentences}}}}} \right)} + 3.12$
Automated Readability Index	$4.71\left( {\frac{{{\text{letters}}}}{{{\text{words}}}}} \right) + 0.5\left( {\frac{{{\text{words}}}}{{{\text{sentences}}}}} \right) - 21.43$
FORCAST	$20 - \left( {\frac{{\# \;{\text{of}}\;{\text{single}}\;{\text{syllable}}\;{\text{words}}\;{\text{in}}\;150\;{\text{word}}\;{\text{sample}}}}{10}} \right)$
New Dale and Chall Index	$0.0496 \times \left( {\frac{{{\text{mean}}\;\# \;{\text{of}}\;{\text{words}}}}{{{\text{mean}}\;\# \;{\text{of}}\;{\text{sentences}}}}} \right) + 0.1579 \times \left( {\frac{{{\text{unfamiliar}}\;{\text{words}}}}{{{\text{mean}}\;\# \;{\text{of}}\;{\text{words}}}}} \right) + 0.363$

The purpose of our study was to assess the readability of patient education materials related to pediatric spinal deformity available from leading pediatric orthopedic centers. We hypothesized that, on average, pediatric spinal deformity-related patient education materials from the top children’s hospitals for orthopedic surgery would be written at greater than the sixth-grade reading level.

## Materials and methods

In December 2021, we searched for spine-related patient education materials from the leading pediatric orthopedic institutions based on US News & World Reports rankings for pediatric orthopedic surgery [19]. We searched each institution’s website for patient information and assessed all web pages pertinent to spinal deformity. In centers that had a specific spine section of patient education, articles with keywords including “scoliosis”, “kyphosis”, “lordosis”, and “deformity” were included. On those websites that did not have a specific spine section, all articles were screened for their relevance and spine pathology by one of the senior authors (CM). The patient education resources were then converted into a text-only format to exclude figures, disclaimers, acknowledgements, citations, references and hyperlinks. Reformatted patient education files were then analyzed using ReadablePro 20201 (Readable, Added Bytes Ltd.; Horsham, UK).

### Statistical analysis

Using this software, the following readability scores were calculated: Flesch–Kincaid (FK), Flesch Reading Ease, Gunning Fog Index, Coleman–Liau Index, Simple Measure of the Gobbledygook Index (SMOG), Automated Readability Index, FORCAST, and the New Dale and Chall Readability. Equations used to calculate these scores are listed in Table 1. All of the aforementioned scores, with the exception of the Flesch Reading Ease, provide a score which correlates with the grade reading level associated with the article (e.g. score of 7 equates to 7th grade reading level). A linear regression analysis was employed to generate variance inflation factors, with values ≥ 10 indicating collinearity between various readability scores [20].

Continuous variables were presented as means and standard deviations. Correlations between institutional ranking and FK scores were assessed using a spearman regression. Additional factors including geographic location (urban versus rural), private versus public institution, and use of concomitant multimedia modalities (pictures or videos present on institutions web site versus no media) that may impact institutional readability scores (as determined by FK) were analyzed with independent *t* test and Mann–Whitney tests for parametric and non-parametric continuous variables, respectively. All tests were 2-sided. Analyses were performed with RStudio 2021.09.1 (RStudio, Boston, Mass., USA).

## Results

In total, all 25 of the top 25 pediatric orthopedic institutions listed on the U.S. News and World Report’s website contained online resources for pediatric spinal deformity. We included 57 web pages in our final analysis. Readability scores were calculated for all web pages that were found in our search. A wide spectrum of FK scores was observed, ranging from 5 to 15.2. Notably, only 11 of the 57 web pages assessed included patient information at or below a 6^th^-grade reading level. These web pages were contributed by only 12 of the top 25 (48%) institutions for pediatric orthopedics as listed by U.S. News and World Report. Overall, the mean composite scores were FK score was 9.0 ± 2.7, Flesch Reading Ease 50.8 ± 15.6, Gunning Fog Score 10.6 ± 3.1, Coleman–Liau Index 11.6 ± 2.6, SMOG Index 11.7 ± 2.0, Automated Readability Index 8.6 ± 2.8, FORCAST 11.1 ± 1.1, Dale–Chall Readability Index 6.4 ± 1.4. Average readability scores for each institution can be found in Table 2, sorted by U.S. News and World Report ranking.

**Table 2:** Readability scores for online patient resources regarding pediatric spinal pathology

Hospital rank	Flesch–Kincaid Grade Level	Flesch Reading Ease	Gunning Fog Score	Coleman Liau Index	SMOG index	Automated Readability Index	FORCAST Grade Level	Dale–Chall Readability Score
1	11.5	41.6	14.6	13.1	14.0	11.4	11.3	7.0
2	9.4	48.4	11.6	12.1	11.9	9.0	11.4	6.5
3	14.4	24.9	17.1	15.9	16.1	14.5	12.0	7.7
4	8.8	52.7	11.2	13.1	11.6	9.5	11.4	7.1
5	6.2	65.6	7.5	9.1	9.4	5.6	10.5	5.4
6	6.7	61.8	7.0	10.1	10.1	6.3	10.6	5.5
7	5.2	74.3	7.8	7.8	9.1	4.7	9.6	4.3
8	9.8	40.6	7.8	10.2	10.7	9.3	12.3	7.4
9	6.7	64.7	9.1	9.6	10.2	6.3	10.2	5.2
10	7.8	56.7	9.3	11.0	10.7	7.3	10.9	6.4
11	9.1	45.7	10.6	12.3	11.7	8.4	11.7	7.2
12	7.8	49.4	7.1	10.4	9.5	6.5	11.9	7.7
13	6.2	63.9	6.5	9.2	9.3	5.5	10.5	5.5
14	6.3	68.1	8.3	9.0	10.0	5.9	9.5	5.0
15	8.9	51.1	10.7	11.8	11.5	8.4	11.4	6.5
16	11.7	33.6	12.8	13.9	13.9	10.8	12.1	7.6
17	13.3	15.5	9.8	17.5	13.3	12.4	13.8	9.2
18	8.2	59.3	10.3	11.1	11.4	8.5	10.5	5.9
19	10.3	47.7	12.1	12.1	12.8	10.2	11.3	6.8
20	9.5	47.9	11.9	12.8	12.1	9.3	11.5	5.9
21	9.9	46.9	12.8	12.0	12.8	9.2	11.2	6.4
22	12.6	25.7	12.4	15.4	14.1	11.5	13.1	9.4
23	15.2	8.3	13.5	19.6	15.2	15.0	13.3	9.2
24	13.1	37.3	15.6	14.2	14.9	13.9	11.9	8.2
25	5.2	74.2	7.5	8.1	9.1	4.9	9.6	4.2

When assessing multicollinearity, it was determined that all demonstrated a high rate of collinearity with FK scores (variance inflation factor for each score: Flesch Reading Ease = 47.5, Gunning Fog Index = 23.5, Coleman–Liau = 11.8, SMOG Index 66.3, Automated Readability Index 20.8, FORCAST 22.7, and Dale–Chall Readability Score 13.0). As a result, FK scores were used to analyze the relationship between readability and other institutional factors including ranking, geographic location, and presence of figures or videos.

The average FK score of the 12 institutions that included some form of illustration, figure or video is 9.069 and the average of those that did not was 9.65. Regionally, the average FK score of the 5 institutions from the Northeast is 9.07, the 6 from the South is 10.16, the 8 from the West is 9.50 and the 6 from the Midwest is 8.58. There was no correlation between ranking and FK Scores (rho = − 0.08, *p* = 0.565). There was no significant relationship found for institutional online resource readability based on FK scores and geographic location (rho 0.15, *p* = 0.261), use of pictures (rho = − 0.14, *p* = 0.2938) or videos (rho = − 0.07, *p* = 0.6212).

24 webpages of the 57 (42%) included in the study contained pictures or illustrations and 3 of 57 included videos (5%). 12 of the top 25 U.S. News and World Report ranked institutions featured pictures or illustrations while only 3 included videos in their patient education materials pertaining to pediatric spinal deformity. Every institution has at least one webpage discussing scoliosis. 15 of the 25 intuitions (60%) has some discussion on kyphosis and only 5 of the 25 (20%) has information regarding lordosis.

## Discussion

The internet has become a vital part of daily life, and its role in disseminating health care information to patients is expanding at a rapid pace [13]. With this expansion, it is important to ensure patient education is not only accurate but also at an appropriate reading level. The U.S. adult population is composed of 5% that are illiterate in English, 14% who have below-basic literacy skills, and 29% with basic literacy skills [21, 22]. Further, a similar distribution was found concerning health literacy, with only 22% of patients having basic health literacy [23]. Health literacy has been an ongoing discussion in the orthopedic community, with previous analyses showing that many materials including those from the AAOS itself were written at reading levels too complex for most patients to understand [1]. The goal of the current study was to assess the readability of parent-facing educational materials on pediatric spinal deformity from top pediatric orthopedic institutions.

**Fig. 1:**
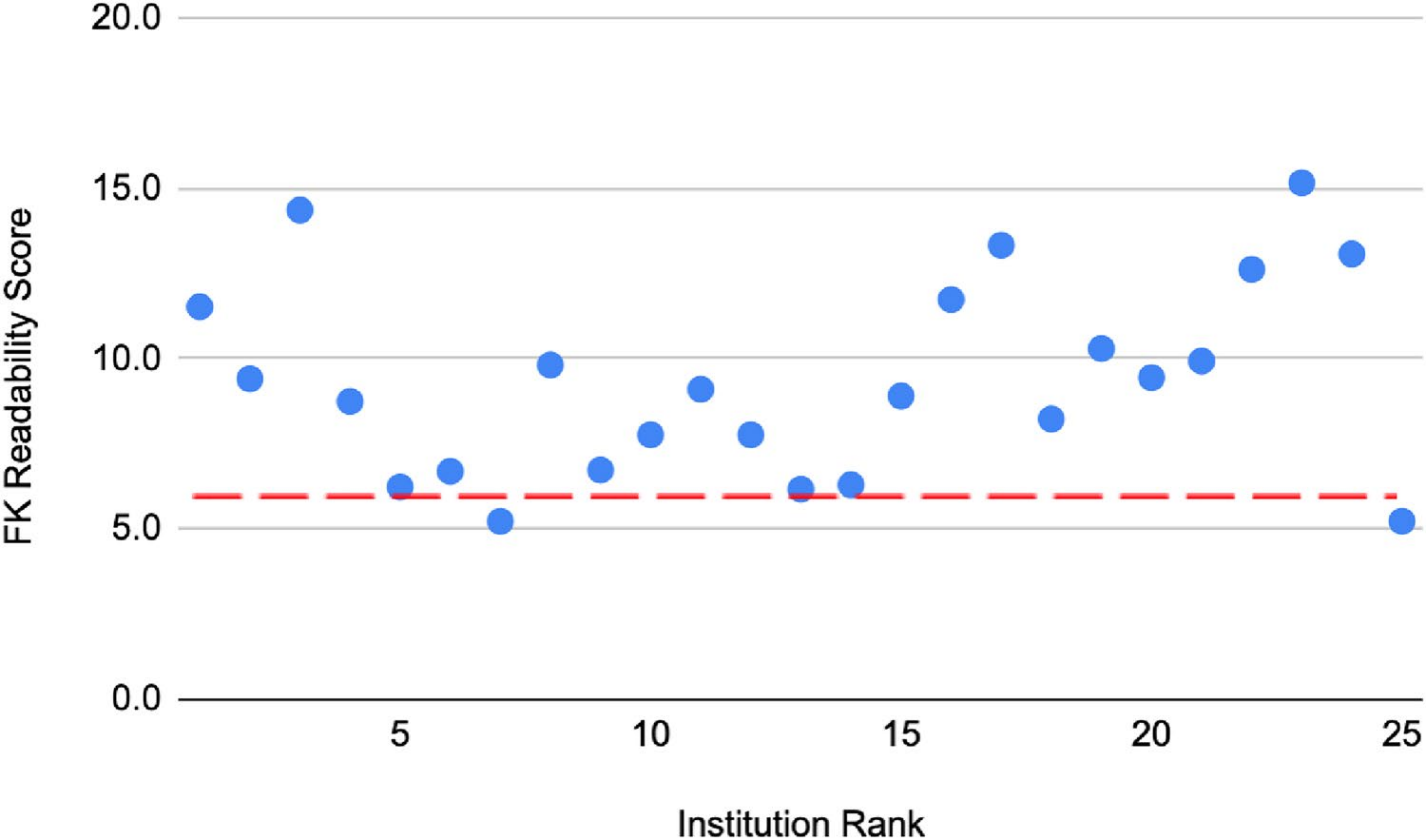
Flesch–Kincaid grade level readability scores for online patient resources for pediatric spinal pathology relative to average united states reading level

The number of institutions featuring educational materials regarding pediatric spinal conditions at a suitable reading level was 12 out of 25 (48%). Furthermore, the average FK reading level of the web pages included in our study was 9.0. Of the 57 web pages included, only 11 (19%) were at or below a 6^th^-grade reading level. We found a high degree of collinearity in all readability scores, indicating there would be little difference when assessing different scores compared to the FK scores that were used. The U.S. News & World Report ranking of these institutions did not have any association with the readability of patient educational materials based on our statistical analysis (rho = − 0.08, *p* = 0.565). These findings are demonstrated in Fig. 1. Geographic location and use of concomitant pictures or videos were also not associated with a statistically significant change in readability levels. Additionally, a severe lack in the inclusion of multimedia in patient educational materials was noted with only 24 of 57 web pages (42%) including figures or illustrations and 3 of 57 (5%) including videos. This represents only 12 of 25 and 3 of 25 institutions that included figures and videos respectively.

Our findings were in concordance with previous studies that assessed the readability of orthopedic patient educational materials [2, 9, 11, 13, 17]. Many of these studies concluded that the majority of orthopedic patient educational materials are at or beyond a 6^th^-grade reading level and have the propensity to confuse both patients and their families. In addition, there were few others who were able to establish an association between institution rank and readability was the study by Parsa et al. which assessed the readability of hip-preservation-related educational materials. This study concluded a weakly negative association between institution rank and readability scores. Institutional-related factors were not found to be statistically significant.

The lack of institutions including multimedia may also be a detriment to patient understanding and comprehension. Videos have been shown in the past to be high quality in content and enhance patient understanding [18]. In the setting of a large differential between readability scores on these web pages and reading level, a video or picture represents a simple way to supplement understanding of pediatric spinal conditions and may be helpful to increase the readability of orthopedic information.

Some other ways to improve the readability of patient resources include choosing words with a single definition, using familiar words, avoiding unnecessary abbreviations or acronyms, and shorter words with decreased complexity [10, 24]. The use of medical jargon and the description of specific anatomical or procedural details can decrease readability. We found the articles with the lowest readability scores overused medical jargon and long complex sentences. Some terms that may be used in place of medical jargon are shown in Table 3. It has been well studied that patient education significantly improves adherence, follow-up and compliance [25]. The internet has become an invaluable educational resource for patients and their families. Top institutions, which are often associated with large academic centers, could improve tremendously by investing in resources and expert educators to help develop patient educational material. Future studies can investigate how the formation of inter-departmental committee boards and communication reviewers at top institutions can elevate the quality of healthcare.

**Table 3:** Identification of commonly used difficult terms related to pediatric spinal pathology

	Term	Alternative
1	Ability	Skill
2	Additional	Added, extra
3	adjacent	Next to
4	Aggressive	Forward, strong, attacking
5	Alteration	Change
6	Anesthetic	Pain reducing
7	Anterior	Front
8	Appear	Seem, come
9	Articular	Joint surface
10	Artificial	Manmade
11	Avascular	Lack of blood supply
12	Benefit	Help
13	Coalition	Joining, union
14	Compress	Squeeze
15	Congenital	Inborn
16	Contain	Have, hold
17	Continue	Keep, keep on
18	Create	Make
19	debilitating	Weakening
20	Debridement	Joint cleaning
21	Deformity	Abnormality
22	Dense	Thick
23	Determine	Decide, figure
24	Develop	Make, grow
25	Difficult	Hard
26	Difficulty	Trouble
27	Ensure	Make sure
28	Evaluate	Check, rate
29	Examination	Check
30	Examine/examination	Check
31	External	Outer
32	External	Outer
33	Fracture	Break
34	Frequently	Often
35	Function	Act, role
36	Identify	Name, find
37	In many cases	Mostly, most of these, often
38	In some cases	At times, sometimes
39	Incorporating	Joining
40	Initial	First
41	Internal	Inner, inside
42	Internal	Inner
43	Known as	Called, named
44	Locate	Find
45	Location	Place
46	Maintain	Keep, support
47	Monitor	Check, watch
48	Multiple	Many
49	Necessary	Needed
50	Opportunities	Chances
51	Option	Choice, way
52	Osteonecrosis	Dead bone
53	Participate	Take part
54	Perform	Do
55	Portion	Part
56	Position	Place
57	Primary	Main, first
58	Procedure	Rule, way, method, treatment, operation
59	Program	Plan
60	Rapid	Quick
61	Recommend	Suggest
62	Reduce	Cut
63	Rehabilitate	restore
64	Remain	Stay
65	Require	Need
66	Result in	Lead to
67	Similar	Like
68	Spine	Back
69	Subsequently	After
70	Surgery	Operation
71	Typically	Often
72	Usually	Often

### Limitations

There are several factors that limited our study. While the readability of patient educational materials does not entirely indicate the quality of patient educational materials, it may increase the patient's ability to comprehend medical information. In addition, while there was a high degree of concordance between the readability metrics that were used, they were not entirely in agreement. There is no clear winner in terms of the best readability metric to use and as a result, we used FK scores as that is what other papers have used in the past to assess the readability of patient educational materials.

## Conclusion

This study demonstrates the readability of educational materials meant for pediatric patients or parents provided by the nation’s top pediatric orthopedic institutions has poor readability. It is concerning that so few institutions have resources at an 6th-grade reading level or below. We recommend avoiding the use of medical jargon and removing detailed explanations regarding procedures from these web pages as well as the inclusion of supplemental pictures or videos to enhance readability and patient understanding. This will ensure a higher degree of health literacy and help to tailor patient expectations and ultimately improve outcomes.
